# Inferring malaria parasite population structure from serological networks

**DOI:** 10.1098/rspb.2008.1122

**Published:** 2008-09-30

**Authors:** Caroline O. Buckee, Peter C. Bull, Sunetra Gupta

**Affiliations:** 1 Department of Zoology, University of OxfordTinbergen Building, South Parks Road, Oxford OX1 3PS, UK; 2 Wellcome Collaborative Research Program, KEMRIKilifi 80108, Kenya; 3 Santa Fe Institute1399 Hyde Park Road, Santa Fe, NM 87501, USA; 4 Centre for Tropical Medicine, Nuffield Department of Clinical Medicine, University of OxfordCCVTM, Oxford OX3 7LJ, UK

**Keywords:** pathogen diversity, *Plasmodium falciparum*, serology, network

## Abstract

The malaria parasite *Plasmodium falciparum* is characterized by high levels of genetic diversity at antigenic loci involved in virulence and immune evasion. Knowledge of the population structure and dynamics of these genes is important for designing control programmes and understanding the acquisition of immunity to malaria; however, high rates of homologous and non-homologous recombination as well as complex patterns of expression within hosts have hindered attempts to elucidate these structures experimentally. Here, we analyse serological data from Kenya using a novel network technique to deconstruct the relationships between patients' immune responses to different parasite isolates. We show that particular population structures and expression patterns produce distinctive signatures within serological networks of parasite recognition, which can be used to discriminate between competing hypotheses regarding the organization of these genes. Our analysis suggests that different levels of immune selection occur within different groups of the same multigene family leading to mixed population structures.

## 1. Introduction

Individuals living in malaria-endemic areas never develop complete immunity to infection with the highly diverse malaria parasite, *Plasmodium falciparum*, although they do acquire protection against clinical malaria after a certain period of exposure and appear to mount a protective response against the more severe forms of the disease after relatively few infections (Gupta *et al*. 1999). The acquisition of clinical, but non-sterile, immunity is still poorly understood; however, it seems to rely on cumulative experience of infection by many antigenically different parasite isolates. An understanding of the antigenic structuring of the parasite population is therefore vital for the design of vaccination and other control programmes, and although large amounts of sequence data from *P. falciparum* antigen genes are currently being generated, a link between genotype and phenotype remains elusive. Here, we analyse serological (i.e. phenotypic) data using a novel technique to address competing hypotheses about parasite population structure, and we discuss our results in relation to current sequence data.

Children growing up in malaria-endemic regions exhibit a gradual accumulation of protective antibodies to different variant surface antigens (VSAs) expressed on parasite-infected erythrocytes. The most well-characterized VSA is PfEMP1 (*P. falciparum* erythrocyte membrane protein 1), believed to be a major target of naturally acquired immunity (Bull *et al*. 1998). Each parasite genome contains approximately 60 *var* genes encoding different PfEMP1 variants (Gardner *et al*. 2002), and these are expressed sequentially in a mutually exclusive manner during infection. *Var* genes exhibit extremely high levels of diversity on a population level. This appears to be due to both sexual recombination in the mosquito and frequent ectoptic recombination between *var* genes on non-homologous chromosomes (Ward *et al*. 1999; Freitas-Junior *et al*. 2000; Taylor *et al*. 2000). Attempts to classify the vastly diverse *var* genes into meaningful groups based on genomic data have led to the identification of approximately six different *var* groups based on upstream promoters, direction of transcription and chromosomal position (Kraemer & Smith 2003; Lavstsen *et al*. 2003, 2005; Kraemer *et al*. 2007; Kyes *et al*. 2007). Another study analysing small *var* sequence ‘tags’ from wild isolates has shown that the proportional representation of these *var* fragments from different groups (using a slightly different sequence-based classification system) appears to be maintained between different parasite genomes, although their expression patterns in different hosts vary considerably (Bull *et al*. 2005). Furthermore, analysis of whole *var* repertoires from three sequenced genomes (Kraemer & Smith 2003; Kraemer *et al*. 2007) and a considerable number of *var* sequence tags from wild isolates has revealed a recombination hierarchy that constrains recombination within different groups to some extent (Bull *et al*. 2008).

Simple mathematical models predict that variant-specific immune responses to *P. falciparum* may lead to a parasite population that is structured into distinct antigenic types, or strains, with non-overlapping repertoires of *var* epitopes by means of immune selection (Gupta *et al*. 1996, 1998). Assuming that the parasite population is composed of discretely circulating strains of varying prevalence and virulence explains several features of malaria epidemiology, including the rapid acquisition of protection against severe disease. This ‘discrete strain’ hypothesis has proved contentious, however, and competing hypotheses range from those suggesting that *var* genes are completely randomly distributed, to those arguing that significant but variable overlap exists between parasite *var* repertoires (Giha *et al*. 1999; Chattopadhyay *et al*. 2003). Unfortunately, testing these ideas remains extremely challenging for several technical reasons. First of all, the locations of antigenic epitopes within the majority of *var* genes remain elusive (although see Dahlbäck *et al*. (2006) and Andersen *et al*. (2008) for bioinformatic analysis of epitope regions within the conserved *var* gene associated with placental binding in pregnant women). Furthermore, *var* diversity makes the design of universal primers problematic, so most sequencing projects focus on the analysis of small sequence fragments rather than whole genes. It is important to note here that simply observing some level of sequence conservation within certain regions of particular *var* gene families does not contradict the discrete strain hypothesis. Understanding the results of these projects is also hampered by the complex and variable expression patterns of *var* genes during infection and *in vitro*. Serological data, by contrast, provide direct information about relationships between the expression of antigenic epitopes and host responses. Here, we attempt to develop a set of tools to dissect these data with a view to extending these methods to eventually allow us to link the genetic structure of *var* sequence fragments to patterns of parasite recognition among patients from endemic regions.

Numerous studies comparing the antibody responses of hosts to their own (homologous) and others' (heterologous) parasites have addressed the question of population structure at the serological level by exploring levels of cross-reactivity between isolates (Marsh & Howard 1986; Forsyth *et al*. 1989; Iqbal *et al*. 1993; Chattopadhyay *et al*. 2003). The patterns of parasite recognition generated by these comparisons are generally presented in the form of a chequerboard of agglutination assays, measuring the difference in antibody titre at the time of acute disease and during convalescence (see appendix A). The chequerboard from the largest study of this kind, involving Kenyan children (Bull *et al*. 1999), is shown in figure 1*a*. Serological studies of this kind have concluded that patients tend to generate antibodies primarily to the VSAs of their own (homologous) parasite, although some cross-reactive responses have been noted (Bull *et al*. 1999; Chattopadhyay *et al*. 2003) and certain parasites are particularly well recognized by heterologous sera (Bull *et al*. 1999, 2000; Nielsen *et al*. 2004). These observations of both isolate-specific and cross-reactive immune responses have not been satisfactorily linked to the competing hypotheses about parasite population structure described above, however, partly because the relationships between cross-reactive responses are difficult to examine quantitatively using the chequerboard representation of the data.

In order to analyse these patterns of parasite recognition within the Kenyan data described above (Bull *et al*. 1999), we use a novel network approach rather than the traditional chequerboard framework. Here, each patient and corresponding parasite isolate is represented as one node within a network, with positive agglutination scores represented as a directed edge between nodes. Edges directed into a node correspond to recognition of the parasite, whereas edges directed out of a node indicate an agglutinating antibody response by the patient (see appendix A for more details). Figure 1*b* shows networks corresponding to the chequerboards in figure 1*a*. Note that the network showing increasing antibody titre, which we assume is generated by the current infection and will hereafter call the ‘response’ network, is simply the difference between the convalescent and acute responses. We use the structure of these networks, in conjunction with simple stochastic models, to illustrate the idea that serological data contain indirect information about the expression patterns and population structure of *P. falciparum var* gene repertoires. We provide evidence for a hierarchy of infection with different parasites among Kenyan children, and propose a *var* repertoire structure that represents an intermediate between a random distribution and the discrete strain hypothesis described above.

## 2. Structural characteristics of Kenyan serological networks

To examine the characteristics of the Kenyan serological networks (figure 1*b*), various metrics of network structure such as degree distribution (the number of edges directed into, *k*_in_, and out of, *k*_out_, nodes), reciprocity and transitivity, and the prevalence of local triad motifs (relationships between sets of three nodes), were compared with randomly generated networks with the same network density. Reciprocity measures the symmetry of two patients' responses, or the extent to which responses towards heterologous isolates are reciprocated (i.e. if patient A recognizes patient B's isolate, does patient B recognize patient A's isolate?). Transitivity relates to the ‘clustering’ of responses or prevalence of triangles in the network, i.e. whether two positive responses to the same isolate tend to occur between patients that also recognize each other's isolates. These normalized measures are standard network metrics and allow for direct comparisons between the acute and response data (see appendix A for more details).

Both networks were significantly different from random expectations for many of the metrics examined, and showed structural features associated with an interesting ‘source–sink’ pattern of parasite recognition. Table 1 illustrates these features. Both had a significantly lower level of reciprocity and a higher level of transitivity than expected, for example, and were associated with a significantly higher variance in both *k*_in_ and *k*_out_ as compared with the random networks. In other words patients tended to show both acute and increasing responses to many or few parasites, and particular parasites were either responded to very commonly or very rarely. It is important to note that the interpretations of the acute and response networks are fundamentally different. Acute networks correspond to the existing recognition of parasite isolates at the time of sampling, and we make the assumption that this reflects the previous exposure of patients to particular antigenic determinants. By contrast, response networks are generated by subtracting the pre-existing ‘acute’ response from the ‘convalescent’ response detected 3 weeks later, and represent antibody titres that are stimulated by the presence of particular antigens during current infections.

Our analysis showed that *k*_out_ for the response network was significantly positively correlated with multiplicity of infection, or the number of distinct parasite clones identified in the patient (*R*^2^=0.45, *p*=0.022), whereas *k*_in_ was not (*p*=0.609). This is expected since patients harbouring more parasite genotypes are exposed to a larger *var* pool during infection, generating increasing antibody responses to a broader range of parasites than patients with fewer parasites. The large range of *k*_in_ values, on the other hand, can be attributed to three isolates (numbered 1026, 1029 and 1032 in the original study) which have *k*_in_ values of 5, 5 and 6, respectively (all other isolates have *k*_in_ values of 0 or 1). Although these three isolates have been previously identified as being ‘commonly recognized’ by acute sera (Bull *et al*. 1999), and display high *k*_in_ values within the acute network accordingly, their role in generating the high variance within the response network requires a different explanation and will be discussed further below.

Figure 2 illustrates the importance of different types of triads occurring in each network relative to 10 000 randomly rewired networks, with the *y*-axis representing the significance of the triad motif with respect to its normalized prevalence (see appendix A). Bars reaching above the 0.5 line shown in the figure are significantly different from random expectations, in accordance with the methods described by Milo *et al*. (2002, 2004). Both serological networks are enriched in triads associated with the source–sink structure described, such as triads 4 and 5 (two edges directed out of a node or into one node, respectively). This type of analysis has previously been applied to many different types of networks, from food webs to regulatory networks to internet connections (Milo *et al*. 2002, 2004). Interestingly, the enrichment of feed-forward loops (triad 9) observed in our acute network has been analysed theoretically and experimentally, and can be found among networks that require or exhibit hierarchical control structures such as social, gene-regulatory and information-processing networks (Milo *et al*. 2002, 2004; Mangan *et al*. 2003; Dekel *et al*. 2005; Cooper *et al*. 2008). We show in §3 that, in contrast to these static regulatory networks, the feed-forward motif in the acute serological network reflects a dynamic hierarchical process of infection.

## 3. Evidence for an infection hierarchy

It has previously been suggested, based on the negative correlation between a host's age and the frequency of recognition of his or her parasite, that there may be a hierarchy of infection with different parasite types (Bull *et al*. 1999). To determine what effects such an infection hierarchy would have on the expected structure of an acute serological network (reflecting previous exposure rather than current infections), simulations were performed in which hypothetical networks were generated assuming either a random or ordered infection process. Infection histories, which can also be thought of as host antibody repertoires, were generated for each of 100 hosts assuming varying levels of exposure. Parasites were assumed to be discrete antigenic types, for simplicity, and infection histories were either chosen randomly from a circulating pool of types or built up with a pre-defined order. Each infecting parasite was then compared with the infection history (or antibody repertoire) of each host, to create a hypothetical acute serological network. Ten thousand simulations were run for each of the random and ordered models, and compared with randomly rewired networks of the same density.

The ordered infection model yielded different network structures from random expectations, whereas the random infection model did not. The ordered model produced networks with extremely low reciprocity, high transitivity, high variance in *k*_in_ and *k*_out_, and a significantly higher density of ‘feed-forward’ loops than expected by chance. Table 2 illustrates the difference between the randomly rewired networks and those simulated under the two different infection processes, as well as the striking structural similarities between the networks generated by the ordered model of infection and the acute network. Intuitively, one would expect these characteristics from a hierarchical infection process. As an example, a patient infected by an isolate ‘higher up’ (for example isolate A) in the hierarchy would recognize one ‘lower down’ (isolate B), but this would not be reciprocated (hence low reciprocity). In addition, the patient infected by isolate A would recognize all isolates below B (e.g. isolate C); the patient infected by isolate B would therefore also be expected to recognize isolate C. Thus, the feed-forward loop A→B→C and A→C is found at high densities and transitivity is high.

The structural characteristics of the acute network are therefore consistent with a hierarchical infection process occurring among these patients, as previously hypothesized (Bull *et al*. 2000). Thus, the immune system of the host (correlating with age in this case) seems to select for the expression of particular antigenic determinants, and children encounter serological variants in a non-random order as they grow up in areas such as Kenya.

## 4. The effects of population structure, infection length and *var* expression patterns on response network structure

We were surprised to find that the response network also exhibited a distinctive hierarchical source–sink structure (see table 2). Unlike the acute networks, edges within response networks are not related to previous infections but rather indicate shared antigenic determinants between currently infecting parasites from different patients. Since the expressed *var* sequences at the time of parasite sampling will be dominated by only the most recently expressed *vars*, the frequency of heterologous immune responses (i.e. those of one child towards the parasites from another child) will depend on (i) the extent of *var* repertoire overlap between isolates and (ii) the order in which repertoires are expressed. Simple models were used to generate hypothetical response networks, and compared with the observed network in order to test three hypotheses concerning *var* repertoire structure and expression patterns. Hosts were infected with parasites defined by 60 *var* genes, and assigned an infection length corresponding to the proportion of their parasites’ *var* repertoire that they experienced during infection. Networks were generated by comparing each host's isolate, which was assumed to be expressing only the last gene in the sequence of *vars* expressed, to every other host's antibody repertoire generated during their current infection. Figure 3 shows a schematic of the model set-up for one such comparison.

Three different simplified population structures were examined, as well as the effects of random or ordered expression of different *var* genes. The population structures modelled were as follows: *var* repertoires could be (i) discrete with non-overlapping combinations of *var* genes, in accordance with the theoretical models of Gupta *et al*. (1996, 1998) described above; (ii) randomly drawn from a global pool of circulating *var* genes, assuming repertoires have been randomized by recombination; or (iii) an intermediate structure combining both these hypotheses, in which each parasite genotype contains a few ‘common’ *var* genes that are expressed early in infection (Lavstsen *et al*. 2005) and are discretely structured due to strong immune selection, and many ‘rare’ *var* genes drawn randomly from a global pool (this hypothesis is based on ideas generated by Bull *et al*. (1999, 2000) see electronic supplementary material for further explanation). For each population, it was then assumed that *var* gene expression occurred either in a particular order or randomly (see appendix A for more details).

To find the models that best approximated the Kenyan response network, we measured network density, transitivity and reciprocity, the number of components formed, and the variance in *k*_in_ and *k*_out_, since these seemed to define the distinctive structure of the data. By comparing the effects of highly simplified hypothetical population structures on expected network structures, we hoped to gain insights into the population structures leading to the observed serological data.

## 5. Support for a partitioned *var* repertoire structure

Figure 4 shows the performance of the three population models; for each structural characteristic, the heights of the bars indicate the parasite diversity at which the model coincides with the observed response network. Absent bars indicate that the model population never reached the value of the parameter observed in the data. Notice that these results are for the best fit parameters for the models in each case (see electronic supplementary material for more comparisons between populations). For the discrete population model, realistic densities and levels of variance in *k*_in_ and *k*_out_ were reached when the parasite population was made up of only seven non-overlapping ‘strains’, although the reciprocity, transitivity and number of components for this population were all extremely high. For populations in which *var* repertoires were drawn randomly from a global pool, only the reciprocity and variance in *k*_in_ reached levels comparable to the data, although this occurred at very different *var* pool sizes (1209 and 532, respectively). The network densities were far below those of the data, and none of the other metrics came close to the values observed. For the best fit intermediate model, however (see figure 4), all six structural characteristics measured coincided with the data when parasite *var* repertoires were defined by a discrete set of *vars* chosen from one of seven non-overlapping strains, and a larger set of *vars* drawn from a global pool of between 260 and 591. The intermediate model also showed features characteristic of the real network over a much larger parameter range than the other two models.

For all three populations, the models most compatible with the data were those with ordered rather than random *var* expression. Without ordered *var* expression, no source–sink structure occurred and transitivity remained low for all three populations—thus some of the features that the response network shared with the hierarchical infection model described in §4 are in this case due to hierarchical expression patterns. It is important to note that variable infection lengths are also important in generating the structures observed in the real network (see electronic supplementary material).

The differences in the compatability of the model population structures with the data can be clearly observed by visualizing the networks in figure 5. For the discrete population, as the diversity of the pathogen population increased, the networks fragmented into different components corresponding to clusters of hosts infected by the same strain (figure 5*b*). Reciprocity remained very high as the number of strains increased, and although the source–sink structure was apparent when *var* expression was ordered, it was weak due to the different components. The random population model showed the same fragmentation as the global *var* pool increased, and there was no source–sink structure observed (figure 5*a*). Densities fell rapidly below that of the observed network—figure 5*a* shows the densest network for this population, for example (assuming a *var* pool of only 100 variants). Of the three population structures, only the intermediate model showed distinctive source–sink structure, low reciprocity, low numbers of cliques and high transitivity. This occurred because many parasites shared the common *var* genes expressed at the beginning of infection (assuming ordered *var* expression), but not those expressed later on, which were randomly drawn from a large global pool. Thus, the isolates taken from those patients with very short infection lengths were recognized by almost all other patients in a non-reciprocal fashion. As seen in figure 5*c*, this led to a pronounced source–sink structure that was focused on these isolates. This finding is consistent with the Kenyan data and provides an intermediate hypothesis between the strain theory of malaria transmission and the arguments that *var* repertoires are randomized. These model population structures are highly artificial, of course, but they do indicate the types of dynamics and population structures that are expected to lead to different types of networks.

## 6. Conclusions

We have shown that serological networks can be used to help understand the population structures and infection dynamics of malaria infections. Given the difficulties inherent to directly identifying strains of *P. falciparum*, and our lack of understanding of within-host dynamics of important antigenic determinants such as the *var* genes, we believe that analysing serological data in this way offers a useful approach to inferring these structures. Our analysis of the Kenyan data suggests a hierarchical structure not only in terms of the infection dynamics of different parasites (the acute network) but also in terms of *var* gene ordering and expression within genotypes (the response network).

We propose that there are different levels of immune selection occurring on different groups of *var* genes, which could lead to the intermediate *var* repertoire structure described above. Here, relatively common *vars* will be structured into discrete, non-overlapping combinations by immune selection, since they are expressed first and by many isolates, and only these restricted combinations will be observed. This hypothesis is compatible with the observation that the group A *var* family appears to be relatively restricted, and it will be testable once the epitope regions of these *vars* have been elucidated (Jensen *et al*. 2004). The relative restriction of diversity among the common genes may also be explained by functional constraints with respect to binding particular host receptors—it has been suggested that commonly recognized *var* genes are optimized for rapid growth among non-immune hosts (Bull *et al*. 1999), also explaining the apparent association of specific subsets of *vars* with severe disease (Rottmann *et al*. 2006; Kyriacou *et al*. 2007). Under our hypothesis, the remaining *var* genes within each genome are not under the same immune selection pressure and therefore do not show the same discrete structure, and will be relatively randomly distributed between genotypes. We conjecture that these *var* genes may also be less functionally constrained, accounting for their diversity.

Bull *et al*. (2008) have generated networks of *var* gene fragments, in which each gene fragment is a node and edges connecting genes are exact sequence matches (see figure 6). This network of *var* sequences forms two main clusters characterized by dense within-cluster links and loose between-cluster connections. The smaller lobe appears to be primarily made up of Group A *var* genes, which form a dense ‘polymorphic block-sharing group’, whereas the larger lobe appears to be composed of both B and C *vars* (based on the classification system of Lavstsen *et al*. (2003) and Kraemer *et al*. (2007)). The recombination hierarchy evidenced by this structure is consistent with our models. We suggest that if the subset of *vars* in the group A block-sharing group is expressed often and is highly immunogenic, immune selection may act to structure it into discrete strains. By contrast, the majority of *var* genes in each repertoire (belonging to groups B and C) may not be expressed to the same extent and would not be under the same level of immune selection. The effects of this type of partitioned *var* repertoire on the overall level of *var* diversity and the development of immunity to disease have recently been explored theoretically (Recker *et al*. 2008). These models showed that the partitioning of *var* repertoires not only limited the level of diversity achievable through recombination, but could also explain the rapid acquisition of immunity to severe disease despite the vast diversity of *var* sequences observed. A better understanding of the link between *var* groups and disease phenotype is needed to confirm the implications of a recombination hierarchy for patterns of immunity and disease, however.

Our analysis also supports the idea that *var* gene expression patterns within hosts are ordered, to some extent, rather than random. Although theoretical models (for a review see Frank & Barbour 2006) and empirical studies (Horrocks *et al*. 2002, 2004) have shown that some level of ordering of expression of different *var* genes is probably needed to maintain antigenic variation within the host, others have concluded that *var* genes are expressed randomly (for example Fernandez *et al*. 2002). The hierarchical structure of the response network, and the fact that low reciprocity between heterologous responses was consistently observed in these data, suggest some intrinsic ordering in expression is likely. The role of the host immune system in the orchestration of these expression patterns is not known, however, and probably plays an important part in determining the order of appearance of different variants (Recker *et al*. 2004).

The diversity of the *var* genes and their complex expression patterns within hosts will continue to hinder attempts to understand their population structure. We believe new methods of analysing serological data are needed, particularly while we remain unable to link sequence and phenotype, to provide insights into the underlying population structure of the malaria parasite.

## Figures and Tables

**Figure 1:**
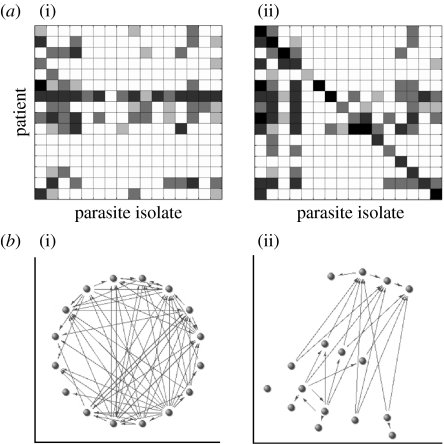
(*a*) A chequerboard of agglutination assays from patients in Kenya. Serological responses of patients (on the *y*-axis) to different parasite isolates (on the *x*-axis). The parasites are presented in the same order as the patients, such that the diagonal of the matrix corresponds to homologous patient–parasite pairs. (i) The acute responses to parasites (the parasite and serum are isolated upon admission to hospital), and (ii) the responses 3 weeks later. The darkness of the boxes corresponds with the strength of the immune response, in terms of the frequency and size of agglutinates formed on a semi-quantitative scale from 0 to 4 (see Bull *et al*. (1999) for details). (*b*) Networks corresponding to the agglutination profiles shown in (*a*). Note that these are (i) acute and (ii) increasing responses rather than acute and convalescent, since we are interested in increasing antibody titres. Each node corresponds to a patient–parasite pair. Positive recognition of parasites is represented by arrows from the patient towards a parasite. We have not included homologous responses.

**Figure 2:**
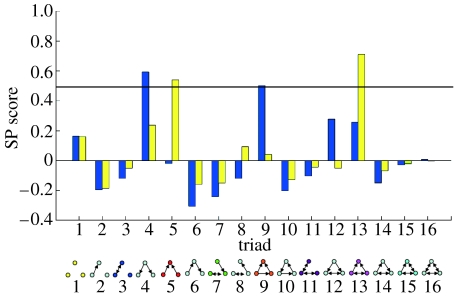
The relative importance of different structural triads in the serological networks. The SP score is calculated as in Milo *et al*. (2002, 2004), which measures the relative importance of different triad types. Here, an SP score above 0.5 represents a significant departure from random expectations. The key shows which triads are referred to. Blue bars, acute; yellow bars, response.

**Figure 3:**
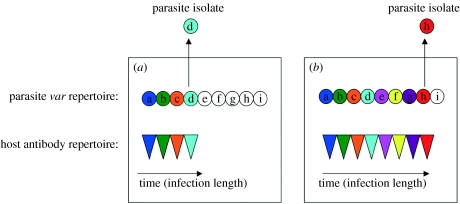
A schematic of the model set-up. In this case an ordered expression pattern and two hosts infected by the same parasite, to illustrate how even this scenario may lead to non-reciprocal responses. Parasite *var* gene repertoires are shown as circles, with different variants having different letters. Colours indicate which variants have been expressed during infection (in this case the parasite has expressed less of its repertoire in (*a*) Host 1 than in (*b*) Host 2). Host antibody repertoires are triangles corresponding to the *vars* expressed during infection. Isolates are assumed to be expressing the final *var* in the sequence of *vars* expressed during the course of infection. In this example, Host 1 will not recognize the isolate from Host 2, since it has not yet experienced that variant from its own parasite's repertoire. Host 2 will recognize the isolate from Host 1, however.

**Figure 4:**
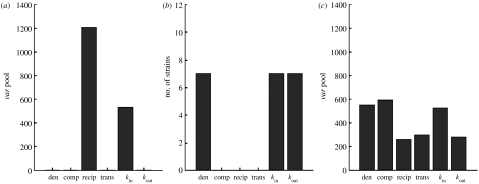
The correspondence of the three model population structures with data. For the six metrics relevant to the increasing network, the heights of the bars indicate the parasite diversity estimated for each population ((*a*) random, (*b*) discrete and (*c*) intermediate) when the model intercepted the observed value. Note that only the intermediate model matches the data for all six metrics.

**Figure 5:**
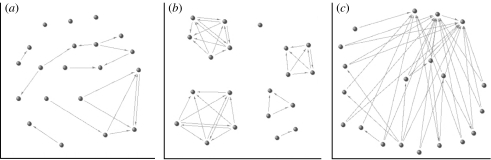
Examples of networks generated by the three models of repertoire structure. (*a*) Assuming a random distribution of *vars*, the network has none of the structural features associated with the data. (*b*) Under the discrete model, the network is fractured into small, dense cliques. (*c*) The intermediate population structure produces networks that have a small number of nodes with many edges directed into them but few out, as observed in the real response network.

**Figure 6:**
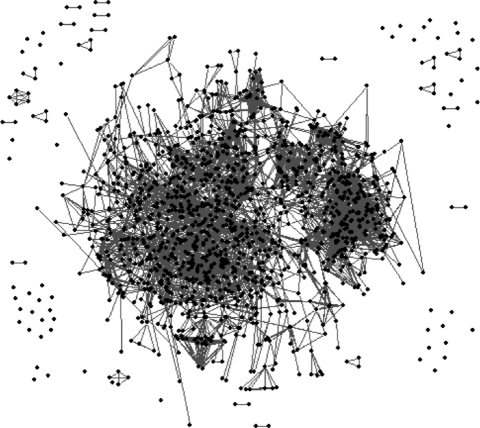
A network of *var* sequence fragments from Bull *et al*. (2008). *Var* sequence tags from the DBLα region from several wild isolates from Kilifi, Kenya, as well as laboratory strains and homologues from the *Plasmodium reichenowi* genome are represented as nodes in the network. Edges between nodes represent exact amino acid matches at one of four polymorphic regions, and are assumed to reflect recombination events.

**Table 1:** Structural characteristics of the serological networks. (See appendix A for the derivation of the metrics. Entries that are asterisked are significantly different from random expectations with *p*<0.05.)

	density	components	reciprocity	transitivity	var *k*_in_	var *k*_out_
acute	0.300	1	0.206^*^↓	0.629^*^↑	12.8^*^↑	27.2^*^↑
increasing	0.104	2	0.080^*^↓	0.250^*^↑	3.2^*^↑	2.9^*^↑

**Table 2:** A comparison of structural features of the model results and the observed networks. (For each of 5 metrics, the directions of the difference between model results or real networks and 10 000 randomly rewired networks of the same density are shown. Boxes with arrows are significantly different from randomly rewired networks in the direction shown. See electronic supplementary material for more results.)

	reciprocity	transitivity	var *k*_in_	var *k*_out_	ffd triads
random model	—	—	—	—	—
ordered model	↓	↑	↑	↑	↑
Kenyan acute	↓	↑	↑	↑	↑
Kenyan increasing	↓	↑	↑	↑	—

## Appendix A

### (a) Agglutination assays and network generation

These assays take advantage of the fact that antibodies present in host serum can bind two identical epitopes at once. When antibody binding occurs on different infected red blood cells expressing the same PfEMP1 variant, the cells clump together into ‘agglutinates’, which can be analysed by means of microscopy based on their size and prevalence (Bull *et al*. 1998). Parasites are isolated from patients upon admission to hospital, representing the acute stage of infection (generally, hosts have not generated antibody responses to their infecting isolate at this stage). The ability of patients' sera to form agglutinates is then measured for every isolate at this acute stage and also a few weeks later, at the convalescent stage of infection. During this period patients generate antibodies primarily to their own parasite, resulting in the diagonal stripe of increasing, homologous agglutination scores observed in the convalescent chequerboard in figure 1*a*. We did not use data from patients who were given a blood transfusion when generating the response network, since the source of the blood was likely to be a semi-immune adult with a large antibody repertoire.

The use of directed networks to analyse relationships between individuals has a long history in sociology and has begun to be adopted by other areas of natural science. As a result, many metrics and ways of analysing network structures are available, and can be used to test hypotheses about complex systems. We wanted to use network theory to quantitatively analyse the structure of cross-reactive immune responses between patients, since this has not been explored systematically in the past. In our networks, the nodes represented both the parasite and the patient it was isolated from. We ignored the relative strengths of agglutination when generating networks from these data and instead used a simple binary measure (i.e. positive or negative response, increasing or not increasing response), since the relative differences in scores are likely to reflect inherent differences between patients rather than differences between parasite epitopes.

Analysis was performed using the network and social network analysis packages in R (Butts 2007; Butts *et al*. 2008; R Core Development Team 2008) and Matlab v. R2007a (Matlab 1997), and network figures were produced using Agna (Benta 2005).

### (b) Derivation of structural metrics and *Z*/SP scores

Network density is defined as the number of observed edges divided by the total possible number of edges, given the number of nodes. The reciprocity of the network is in this case the ‘edgewise’ reciprocity, defined simply as the proportion of reciprocated edges. Transitivity here is measured as the proportion of potentially intransitive triads that satisfy the constraint: a→b→c⇒a→c (this is the ‘weak’ form of transitivity).

*Z* scores give the prevalence of different triads in the data compared with equivalent, randomly rewired networks (see Milo *et al*. 2002, 2004). SP scores are normalized *Z* scores, representing the relative importance of each triad. The *Z* score for each triad *i* is derived as follows:

$$Z_{i}=(N_{{\text{real}}_{i}}-\langle N_{{\text{rand}}_{i}}\rangle )/\text{std}(N_{{\text{rand}}_{i}}),$$

where $N_{{\text{real}}_{i}}$ is the observed number of occurrences of triad *i* in the data, and $N_{{\text{rand}}_{i}}$ is the number of triads in the randomly rewired networks. Here, for each observed network we generated 10 000 randomly rewired networks.

The SP score for each triad *i* is then derived as

$${\text{SP}}_{i}=Z_{i}/{\left(\sum Z_{i}^{2}\right)}^{1/2},$$

such that the vector of *Z* scores is normalized to give relative rather than absolute significance of different triads.

### (c) Modelling the effect of population structure and expression patterns on hypothetical response networks

The parasite population was generated assuming that each parasite had 60 *var* genes that were: (i) drawn randomly from a global *var* pool (which varied in size between 70 and 2000 *var* genes); (ii) chosen from a pool of one of several strains defined by non-overlapping *var* gene repertoires (the number of strains was varied from 2 to 50); or (iii) in part chosen from non-overlapping combinations of *var* genes (10 genes per genome) and in part drawn from a global pool. For the latter case, the intermediate population, the number of strains and global *var* pool was varied within the same range as the other populations. Hosts were assumed to be exposed to a fraction of their parasite's *var* gene repertoire, drawn from a normal distribution around mean exposure varying between 10 and 60 of the possible 60 *vars*. The effects of differences in infection length were explored by changing the variance of the mean number of *vars* experienced ranging between 2 and 32.

## Supplementary Material

Supplementary material All supplementary material
