# Supplementary material for: Inferring malaria parasite population structure from serological networks
Source: Proc Biol Sci. 2008 Sep 30;276(1656):477–85. doi: 10.1098/rspb.2008.1122 (PMC2581777; doi:10.1098/rspb.2008.1122)
Supplement: Supplementary file 1 [file rspb20081122s05.doc]

**Serological Networks: Supplementary Material**

1. **Structural features of random versus ordered networks and observed serological networks**

The table below gives the means and standard errors of structural characteristics of 10,000 simulated networks under the ordered and random models of infection, plus the observed values for the observed acute and response networks and the means and standard errors of 10,000 randomly re-wired networks of the same density. These are the values used to generate Table 2 in the main text.

|  | **Reciprocity** | | **Transitivity** | | **Variance k_in_** | | **Variance k_out_** | | **Triad 9** | |
| --- | --- | --- | --- | --- | --- | --- | --- | --- | --- | --- |
|  | *Ordered model* | *Random model* | *Ordered model* | *Random model* | *Ordered model* | *Random model* | *Ordered model* | *Random model* | *Ordered model* | *Random model* |
|  | 0.138±0.0003 | 0.274±0.0004 | 1.00±0.00 | 0.325±0.0005 | 889.0±0.6 | 13.1±0.12 | 786.9±0.59 | 112.1±0.55 | 127538±81.9 | 2363±13.6 |
|  | *Observed* | *Simulated* | *Observed* | *Simulated* | *Observed* | *Simulated* | *Observed* | *Simulated* | *Observed* | *Simulated* |
| **Acute** | 0.206 | 0.298±0.0005 | 0.629 | 0.297±0.0002 | 12.8 | 4.2±0.013 | 27.2 | 4.2±0.013 | 187 | 74.1±0.017 |
| **Response** | 0.080 | 0.099±0.0006 | 0.250 | 0.098±0.0005 | 3.2 | 1.4±0.005 | 2.9 | 1.4±0.005 | 3 | 2.5±0.016 |

2. **Justification for intermediate model of population structure**

Bull *et al.* [16] have generated networks of *var* gene sequence tags in which each gene is a node and edges connecting genes are shared exact matches (shown in the figure below). This network of genes forms two main clusters that form dense links internally, and are loosely connected to the rest of the network. Group A *var* genes, which are shown as coloured brown, red, and yellow in Figure 1 below and correspond to groups 1, 2, and 3 in the classification system of Bull *et al.*, 2007) map primarily to the smaller lobe of the network. B and C *vars* (based on the classification system of Lavstsen *et al.*, 2003), on the other hand, map to the larger lobe. It has been shown that Group A *vars* tend to be expressed early in infection (Lavstsen *et al.*, 2005) and this has led to the idea that they may be associated with severe disease and young host age, although there is no direct evidence of this. Bull *et al.* (1999) have shown that variant surface antigens (VSAs) expressed by parasites from younger children are recognized by heterologous sera more frequently than VSAs from older children and adults. We propose that if this subset of *vars* (the smaller lobe) is indeed expressed often and is highly immunogenic, immune selection may act to structure it into discrete strains.

***Figure 1*** *Var network from Bull et al. (2008). Nodes represent var sequence tags from wild Kenyan solates and lab strains, edges between them represent exact matches at one of four “position-specific polymorphic block” regions (PSPBs). Coloured nodes represent var tags belonging to group A, with different colours representing groups 1, 2, and 3 in the classification system of Bull et al. (2007)*.

In contrast, the majority of *var* genes (forming the larger lobe on the left of the network) in each repertoire may not be expressed as frequently and therefore won’t be under the same immune selection. Figure 2 shows a schematic of this idea, with the expected general characteristics of each lobe under our hypothesis of intermediate population structure.

***Figure 2*** *Expected levels of immune selection and var composition of the two lobes of the network under our hypothesis of an intermediate var repertoire structure.*

3. **More results of the population structure models**

Below are comparisons of the three different population structure models, and their fit to the observed data as a function of parasite diversity: the size of the *var* pool in the case of the random population, the number of strains for the discrete population, and the size of the *var* pool for the best fit intermediate model, which was for 7 non-overlapping strains among the discrete portions of repertoires. The figures 3a-e below correspond to the best fit values given in the main text in Figure 4, and although the exact values of parasite diversity are not shown for reasons of simplicity, the aim is to illustrate general relationships between diversity and the parameters of the networks measured.

*Figure 3a) relationship between parasite diversity and network density*

*Figure 3b) relationship between parasite diversity and number of network components*

*Figure 3c) relationship between parasite diversity and network reciprocity*

*Figure 3d) relationship between parasite diversity and network transitivity*

*Figure 3e) relationship between parasite diversity and variance in k_out_ (note that this figure is the same for k_in_).*
